# Simple quantitation and spatial characterization of label free cellular images

**DOI:** 10.1016/j.heliyon.2024.e40684

**Published:** 2024-11-23

**Authors:** Vincent C.J. de Boer, Xiang Zhang

**Affiliations:** Human and Animal Physiology, Department Animal Sciences, Wageningen University, De Elst 1, 6708WD, Wageningen, the Netherlands

## Abstract

Label-free imaging is routinely used during cell culture because of its minimal interference with intracellular biology and capability of observing cells over time. However, label-free image analysis is challenging due to the low contrast between foreground signals and background. So far various deep learning tools have been developed for label-free image analysis and their performance depends on the quality of training data. In this study, we developed a simple computational pipeline that requires no training data and is suited to run on images generated using high-content microscopy equipment. By combining classical image processing functions, Voronoi segmentation, Gaussian mixture modeling and automatic parameter optimization, our pipeline can be used for cell number quantification and spatial distribution characterization based on a single label-free image. We demonstrated the applicability of our pipeline in four morphologically distinct cell types with various cell densities. Our pipeline is implemented in R and does not require excessive computational power, providing novel opportunities for automated label-free image analysis for large-scale or repeated cell culture experiments.

## Highlights

• High-content imaging without fluorescent labels can be effectively used for interrogating cellular biology.
• An automated label-free image processing pipeline for cell number quantification and spatial characterization was optimized.
• Diverse cell types were validated across a broad range of cell densities spanning two orders of magnitude.
• The pipeline does not rely on training data.
• The pipeline is fully implemented in R, taking advantage of R's robust statistical capabilities and tidy syntax.

## 1 Introduction

Collecting data from cell and tissue samples relies more and more on high-throughput and automated optical microscopic imaging. To this end, fluorescent labeling or fixation is often used to extract biological information from microscopic images. However, fluorescent labeling may interfere with intracellular biology, and fixation can only provide terminal readout. In contrast, label-free methodologies, ranging from brightfield, phase-contrast, and differential interference contrast (DIC) to imaging of autofluorescence and Raman-based imaging [1], have the advantage that intracellular biology remains intact, and minimal processing steps are required that can save substantial wet-lab procedural time. Despite the advantage of label-free imaging technologies, label-free image analysis is challenging because it lacks bright foreground signals that are typically produced by experimentally added fluorophores, which improve the contrast between background and foreground signals. To address this challenge, instrumental adjustments have been made that process the transmitted light to generate phase-contrast, high-contrast brightfield or DIC [2]. These sophisticated imaging modalities allow for detailed analysis of label-free images, such as quantitative phase imaging of clinical samples [3], but at the moment are not available for routine automated cell culture monitoring [3].

Alternative to hardware and procedural changes, one can also use computational techniques to extract meaningful information from label-free images. In this study, we focused on the number and distribution of cells in the image since these types of information are routinely required in everyday lab work and directly affect the experimental readouts. Interestingly, scientists often collect these data either through commercial software, by manual assessment, or by using open-source software. Commercial software packages are offered by vendors of microscope set-ups (eg. Imaris, Zen, In carta). Open-source software that is widely used are ImageJ and cellprofiler [4,5], but also the machine learning tool Ilastik is able to provide image segmentation options. However, these options often require manual configuration by the user which can introduce bias or make analysis workflows labor intensive. Previously, deep neural network based tools have shown promise in accurately recognizing and segmenting cells in bioimages [[6], [7], [8], [9], [10], [11], [12], [13], [14], [15], [16], [17]]. However, the accuracy of these methods depends on the size of training datasets and require intensive manual annotation [[18], [19], [20]]. Moreover, to reduce the risk of artefactual outcomes, also manual configuration is often required [21,22], making these methods often not generalizable over multiple cell types and image acquisition modalities.

In the current work, we aimed to provide all scientists with a computational tool that is open source and can be applied to high-content cell imaging microscopy experiments. To this end, we developed an automatic image processing pipeline for cell number quantification and spatial characterization for label-free images. Specifically, our pipeline does not require any training dataset, and uses a number of classical image processing functions to enable cell number quantification based on label-free brightfield images. Furthermore, we applied Voronoi segmentation and Gaussian mixture modeling to improve the accuracy of cell quantification and spatial characterization. We demonstrated the scalability, generalizability, and usability of our pipeline by analyzing high-content brightfield and phase-contrast imaging of four different cell types.

## 2 Materials and methods

### 2.1 Cell culture and microscopy

C2C12 mouse myoblasts and THP1 human monocytic cells were obtained from ATCC (CRL-1772 (RRID:CVCL_0188) and TIB-202 (RRID:CVCL_0006), respectively). C2C12 and THP1 cells were routinely cultured in either DMEM (#11960044) or RPMI 1640 (#31870025) respectively, with sodium pyruvate (1 mM) (#11360039), glutamax (2 mM) (#35050038), penicillin-streptomycin (1 %) (#15140122), HEPES (10 mM, #15630) and FBS (10 %, #10500064). All components were from Gibco, ThermoFischer. For imaging, cells were plated at multiple densities in 96 well plates (Seahorse XF96 cell plates, #103794-100, Agilent). C2C12 cells were plated at 1000 cells/well to 15,000 cells/well, on the day before the image acquisition. For THP1 cells, cells were plated at 5000 to 100,000 cells/well followed by stimulation with 100 ng/ml phorbol 12-myristate 13-acetate (PMA, #P8139 Sigma) for 48 h. After a subsequent rest period for 24h in normal culture media cells were images. Both cell lines were authenticated within 3 years of use in experiments and were tested negatively for mycoplasma.

Wells of the 96-well plates were imaged automatically using a Cytation-1 Cell imaging multimode reader (Agilent) set at 37 °C using the Gen5 (version 3.12) software. For brightfield images, one image per well was captured with an Olympus 4× UPLFLN objective, using user-trained auto-focus and manual exposure settings that were set before imaging of each plate. For nuclei counting, 4 μM of Hoechst (33342, #B2261, Sigma) from a 40 μM stock solution was added 15 min before imaging to each well. Nuclei image fluorescence readings were taken using the same Olympus 4× UPLFLN objective and a 365 nm LED with an EX337/EM447 DAPI filter cube.

### 2.2 LIVECell images

Images for the A172 and A549 cell lines were obtained from the LIVECell-2021 dataset that was downloaded from the AWS S3 bucket following the instructions on the LIVECell Github pages (https://sartorius-research.github.io/LIVECell/) on January 3rd, 2024.

### 2.3 Nuclei-stained image analysis

Fluorescence images from nuclei-stained C2C12 cells were processed and segmented using an approach outlined in Huber and Holmes [23] implemented using EBImage with functional programming in R. First, images were thresholded using a background image that was generated using a 2D convolution function (EBImage::filter2) with a disc shaped brush of 21 pixels in size. This generates a binary image with value 0 for background pixels and value 1 for foreground pixels. To smoothen the binary image, a morphological operation is applied that smoothens the image using a small disc-shaped brush with the size of 3 pixels (EBImage::opening). To be able to segment the images, a watershedding approach was used (EBImage::watershed) followed by a connected sets algorithm (EBImage::bwlabel). For the watershedding procedure of the binary image, first a distance map was generated (EBImage::distmap) that calculates for each foreground pixel, how far it is located from its nearest background pixel. In the segmented images the isolated unique objects were counted.

For THP1 cells, the nuclei counts were less accurate when using the above pipeline, likely because of the heterogeneity in nuclei size and intensity of staining, which was not observed for C2C12 cells. We therefore adapted the above nuclei segmentation with additional procedures from the imager, imagerExtra, and EBImage packages. After the 2D convolutional function (EBImage::filter2), we included a multi-level thresholding filter (imagerExtra::threshML). Moreover, to adjust for background staining and image artefacts, we performed a histogram equalization and removed inhomogeneous backgrounds using a Screened Poisson Equation (SPE). After the multi-level thresholding, we cleaned the image from small speckles and filled circular objects, followed by the same watershedding function that was applied as for the C2C12 cells.

The nuclei counts for the A172 and the A549 were downloaded from the same AWS bucket that was used for downloading the images in JSON format and loaded into R.

### 2.4 Brightfield image cell number quantitation

For segmenting and quantitating the brighfield images, a workflow was designed that consisted of the following steps.

1) Cell size determination
2) Classical image analysis
3) Voronoi segmentation
4) Expectation-Maximization

In detail, the first step was to acquire the cell size by using a R Shiny GUI that allowed us to draw polygons around cells in the image followed by calculating the cell area size in pixels. Twenty individual cells were traced and the mean cell size area, which is the target cell size area, was taken for optimization of the image processing pipeline. In the second step of the workflow, images were subjected to a pipeline of classical image processing steps. Images were first read into R from a local folder followed sequentially by blurring, adaptive thresholding, edge detection, cleaning, and shrinking with an optional pixel growth step.

Image ->

• magick::image_read(.)
• imager::magick2cimg(.)
• imager::isoblur(.)
• imager::ExtraThresholdAdaptive(.)
• imager::clean(.)
• imager::imgradient(.)
• imager::enorm(.)
• imager::grow(.)
• imager::shrink(.)

This sequence of functions generated a mask with pixels that correspond to cell positions. The xy-positions of all connected sets of pixels was considered as a seed for input into the Voronoi segmentation. In the third step, the Voronoi segmentation was performed using the R deldir package with default arguments. After the segmentation, in the fourth step, the generated tiles were used to classify the segments into one of three classes by Expectation-Maximization with functions from the mclust package in R. The class two tiles were defined as the tiles that represent the original cell size (as described in Fig. 3 of the results section). The mean tile area size of the class two tiles was used as parameter to be optimized.

The above sequence of image processing steps, Voronoi segmentation and Expectation-Maximization was iterated over a set of predefined parameters that were the arguments of the image processing steps. In total, a combination of six parameters were optimized that each could be set to 1 to 4 discrete values. Combinations of these parameters were run, generating up to 20000 iterations of the complete processing pipeline. The class two tile area for each iteration were sorted based on lowest distance from the target cell size area and the mean number of cells in the whole image of the top 20 was set as the cell number of that image.

### 2.5 Brightfield image spatial analysis

For spatial characterization of an image, the top parameter set that was closest to target cell size area was used and the xy positions were extracted. The xy-positions were used as input for the dbscan function from the dbscan R package with eps = 40 and minPts = 5. The dbscan clusters that were identified were merged with the Voronoi segmentation data and the 2D plots were generated in ggplot coloring each tile by cluster. The tiles that were identified by mclust as class three were not labeled, since these tiles represent areas without any cells. Also, tiles that were labeled as not being part of a cluster by dbscan were not colored. Within cluster density was determined by taken the total number of objects labeled with the same cluster ID divided by the total tile area of that cluster.

We used the spatstat R package to determine the deviation from complete spatial randomness. For this, first the xy positions were converted to spatstat point patterns, followed by running the quadratcount and quadrat. test functions using a 10 by 8 grid. The Chi-squared values as generated in the quadrat. test function were obtained for each selected image.

Confluency of images was determined by taking the surface area of the class one and two tiles in pixels divided by the total number of pixels in the image. Tile area mean, sd and cv were calculated from the same class one and two tiles.

## 3 Statistics

Comparison of estimated counts from the nuclei-stained images with the estimated counts of the label-free images was done using the cor. test from base R using spearman. The rationale for selecting this test was that the test evaluates the correlation based on non-parametric principle which is particularly suitable for count data. The strength of the association between the nuclei-stained and label-free estimated counts was assessed with the R-squared value. The R version that was used to run scripts and analyze data was R 4.4.0. Relative error was calculated using magnitude-based comparisons with the formula (log(estimated cell number) – log(nuclei))/log(nuclei). Presented images in figures are 1017 × 749 pixels with a pixel representing 1.68 μm, magnifications are cropped from the individual image and always have the same pixel/μm ratio as the orginal image.

## 4 Results

### 4.1 Automated pipeline for label-free image analysis in R

To be able to manage high-throughput image analysis in a fully R based environment with functional programming, we combined existing image processing functions using the image analysis R packages imager, imagerExtra, EBImage and magick. We opted for a programming style using the tidy approach, by applying dplyr's piping capabilities to connect a series of existing image processing and handling functions. For repeating processing pipes on multiple images or multiple parameter inputs the functional pmap from the purr package was used, which was extended to the paralellized functional furr equivalents, offering computing time increases. We tested the computational time of the analysis of a set of ten plates containing 96 brightfield images each (960 images in total) on a Mac laptop with M2 pro processor. Either a 3 step processing (cropping, blurring and adaptive thresholding) or a 6 step processing (with additional erosion/dilation and edge detection) was tested. For both the 3-step and 6-step processing the furrfuture_pmap functional was the fasted with a median processing speed of 86 s and 80 s respectively. The non-parallelized purrpmap, as well as common for-loops took longer, up to 252 s. For all following image processing we used the future_pmap functional for calculations.

### 4.2 Sequential application of classical image processing functions to obtain seed points in label-free brightfield images

**Fig. 1:**
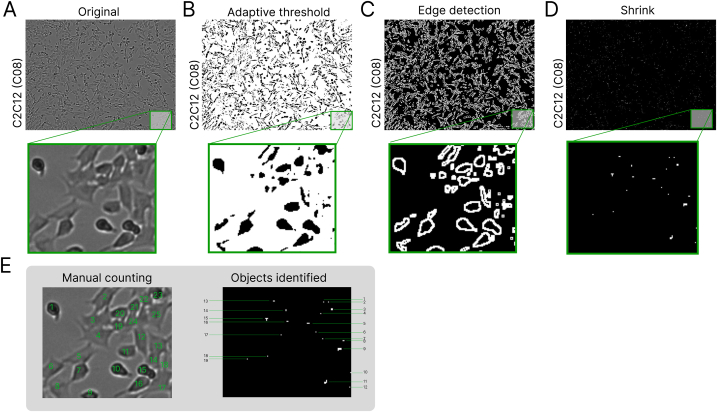
Overview of consecutive image processing steps of a single image of C2C12 cells cultures in a well (C08) of a 96 well plate. A) The unprocessed bright-field image. B) Binary image mask after blurring and adaptive thresholding was performed. C) The result of the edge detection step. D) Shrinking (or erosion) result of the binary image in C. E) Manual identification and counting of individual cells in the original image from A as compared to the objects identified in the image from D. Manual counting results in 23 observations whereas the automatic object identification generates 19 objects. In all images the image and a region of interest is magnified and outlined in green. Presented images are 1017 × 749 pixels with a pixel representing 1.68 μm, the magnification is cropped from the image and has dimensions of 133×114. (For interpretation of the references to color in this figure legend, the reader is referred to the Web version of this article.)

Next, we aimed to extract relevant information from label-free brightfield images with relatively low resolution and where foreground and background are difficult to be distinguished automatically. Rather than shape or other morphological information, our pipeline focused on quantifying cell number and characterizing spatial distribution of cells in an x-y plane. To this end, we needed to identify a set of seed points that indicated parts of cells. For example, when nuclei staining is applied, cells can be observed through highlighted nuclei. However, in label-free brightfield images, there is no staining. To generate seed points, our pipeline leveraged classical imaging process procedures to find objects that can represent part of each cell. As an example, C2C12 myoblast brightfield images (Fig. 1A) were first subjected to blurring and adaptive thresholding to produce a binary image (Fig. 1B), followed by edge detection (Fig. 1C) to select the pixels that have the highest contrast as compared to their neighbouring pixels. In this way, we were able to filter out background pixels and continue only with pixels that belong to the objects of interest. As can be seen in Fig. 1C, outlines of cells were mostly kept, whereas the interiors of cells were not. Moreover, the individual cells were not necessarily distinguishable anymore, because outlines of one cell might connect with neighbouring cells. To be able to still get information for individual objects we reasoned that by dilating (or shrinking) the objects with a defined number of pixels (Fig. 1D), the remainder of the pixels could represent parts of individual cells. Indeed, when counting cells manually in a zoomed area of the image we counted 24 cells and identifying all separated objects computationally in the dilated image gave 19 objects (Fig. 1E). Thus, by applying a number of classical imaging processing functions sequentially in an R data pipeline, we could potentially estimate cell number and approximate their spatial location.

### 4.3 Seed points refinement with voronoi segmentation, mixture modeling, and parameter optimization

**Fig. 2:**
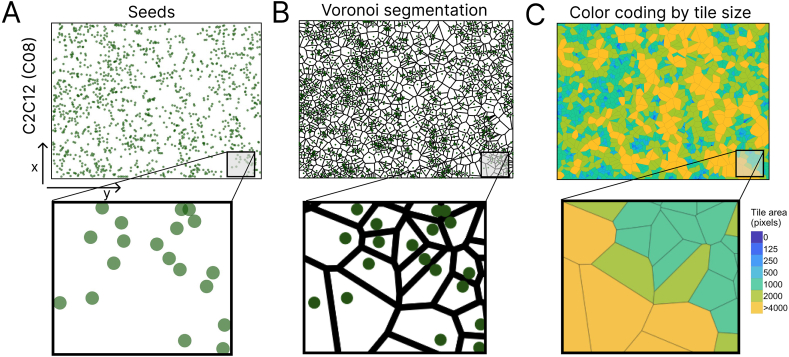
Voronoi segmentation of the representative C2C12 (well C08) image. A) The xy positions (called seeds) of the identified objects from Fig. 1D were plotted in a 2D plane. B) Voronoi segmentation based on the given seeds from A. C) Color coding on a discrete binned scale of each tile based on the area of each tile. (For interpretation of the references to color in this figure legend, the reader is referred to the Web version of this article.)

We noticed that our imaging process procedures often ended up with more seeds than the actual cell number due to redundant seeds from the same cell, Furthermore, depending on the parameter value used in the imaging process functions, different seed points can be generated and lead to different cell number quantification outcome. To mitigate redundant seed points, we first performed Voronoi segmentation based on the seed points from our image processing steps (Fig. 2A). Voronoi segmentation divides a plain into polygons that are generated based on a set of seed points and the distance between a seed point and its neighbouring seeds [24]. We observed that small Voronoi tiles corresponded to higher cell density, whereas large Voronoi tiles corresponded to sparsely populated regions (Fig. 2B). When we colored the tiles for tile area size (as in Marsh et al. [25]), the color mapping clearly demonstrated the local density of cell distributions with large tiles colored in yellow representing areas with few cells and darkblue tiles representing areas with high cell density (Fig. 2C).

**Fig. 3:**
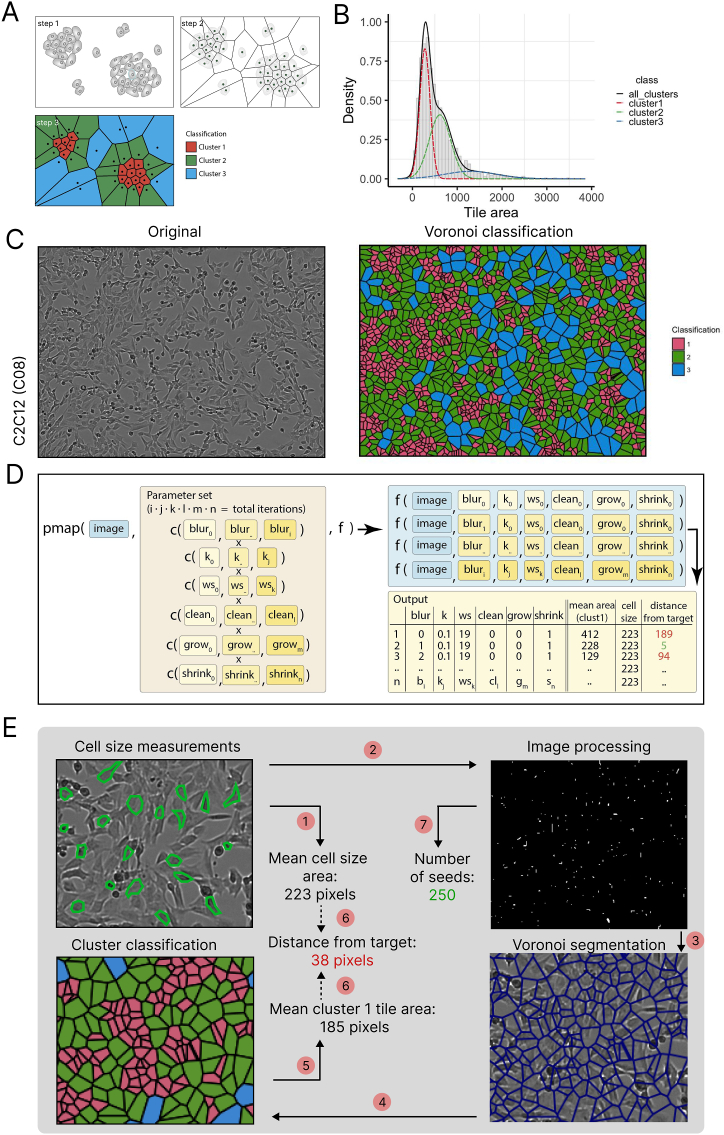
Seed points refinement with mixture modeling, and parameter optimization. A) Illustration of the Voronoi segmentation and cluster definition for mixture modeling of a simulated set of seed points. B) Density distribution of the three clusters in red, green, and blue (dashed lines). With the histogram (binned bars) and density distribution (black solid line) of the whole population of tiles. C) Coloring of the Voronoi segmented C2C12 (well C08) image by cluster number. D) Brute-force grid approach for optimization of the parameters in the classical image processing functions. The dplyrpmap function was used to map a defined parameter set to the image with a function f. The function performs the full pipeline (image processing, Voronoi segmentation and mixture modeling) to output the ‘mean area of cluster 1 tiles’ and the ‘distance from target’, along with each input parameter for each iteration. E) Overview of the whole image processing pipeline. A single image is used as input for the cell size estimation (step 1) (as outlined by the green polygons). In step 2 to 4, the image is processed, segmented and clusters are identified. In step 5, the ‘mean cluster 1 tile area’ is calculated, which is compared to the estimated cell size from step 1 in step 6. This is iterated over the parameters and for the top parameter sets with the smallest distance to target are used. The ‘number of seeds’ (step 7) corresponding to the optimal parameter sets are used as a proxy for cell number in the image. (For interpretation of the references to color in this figure legend, the reader is referred to the Web version of this article.)

To demonstrate how a Voronoi segmentation can be used to segment an image based on seed points, we used a dummy image (Fig. 3A “step 1”), for which we converted the middle point of each cell into the seed point, that was then used for generating a Voronoi segmentation (Fig. 3A “step 2”. This process results theoretically in three types of tiles, either tiles that are small and represents cells in the inner mass of a cluster (cluster 1 cells), cells on the edges of a cluster (cluster 2) and tiles that typically represent areas that do not contain cells or only very few (cluster 3) (Fig. 3A “step 3”). When the Voronoi tile area is used as a proxy for the estimated cell size, and fitted to a Gaussian mixture model, we found that Voronoi tiles were assigned to one of these three clusters. The distribution of tile area showed that it peaked at cluster 1 (Fig. 3B) and the cluster 1 tiles overlapped mostly with cells in dense areas (Fig. 3C). To optimize the parameter values used in image processing, we used a brute-force grid approach so that the distance between the actual cell size area and the mean cluster 1 tile area was minimized (Fig. 3D). To obtain the actual cell size, our pipeline provides a R shiny tool which loads the image and allows users to draw a polygon shape around cells followed by recording the cell size area in pixels. The mean of customized number of (for example, 20) observations was then set as cell size target (Fig. 3E). In the end, the total number of seeds generated based on the optimal parameter values were used as a proxy to indicate the number of cells.

### 4.4 Validation of the procedure

**Fig. 4:**
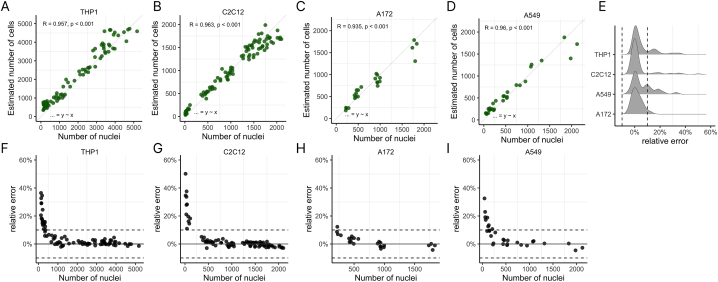
Comparison of results from new label-free image processing pipeline with ground truth Hoechst-stained nuclei counts for four different cell lines. A) THP1 monocytic cells attached to bottom of wells using cell-tak. B) C2C12 myoblasts grown in wells overnight. C) A172 glioblastoma cells at 24 h after plating and 48 h after plating. D) A549 lung adenocarcinoma cells at 24 h after plating and 48 h after plating. E − I) Relative error was calculated using magnitude-based comparisons with the formula (log(estimated cell number) – log(nuclei))/log(nuclei) with the overview of all four cell lines in E). F – I are the relative errors of the data presented in A- D. Each green dot or black dot represent the count or relative error of one image. The dashed line represented the y = x line and the Spearman correlation was reported for each comparison in A- D. The dashed lines in F-I represent the (±) 10 % relative error. (For interpretation of the references to color in this figure legend, the reader is referred to the Web version of this article.)

Now that we set-up an image processing and cell number quantification procedure, we wanted to validate whether our estimated number of cells indeed represent true cell numbers. For this we used two datasets from our own lab (C2C12 myoblasts and THP1 monocytic cells) and two publicly available datasets (LIVECell A172 glioblastoma cells and A549 lung adenocarcinoma cells [12]). These four cell types have distinct morphologial features and were imaged using two distinct high-content imaging instruments each with distinct magnification and resolution. All datasets also included images from cells at a wide range of seeding densities, allowing us to test the scalability of our approach. The nuclei counts for these images, obtained using fluorescently labeling nuclei with Hoechst, were used to represent the actual number of cells. We assessed the cell size area of each individual celltype for each plated cell density and ran the brute-force grid approach on each individual image with matching cell size target. For the THP1 and C2C12 images we observed that the estimated and actual cell numbers have similar magnitude and are correlated with a Spearman correlation of 0.957 and 0.963 was obtained, respectively (Fig. 4A and B). The A172 and A549 image data sets had longitudinal image acquistion on multiple days. We choose the images that were acquired after 24 h and 48 h after start of imaging experiment for the analysis. Similar to our own C2C12 and THP1 images, we observed the estimated cell number and the actual cell number of A172 and A549 have similar magnitude and are correlated, with Spearman correlation of 0.935 for A172 (Fig. 4C) and for A549 images a Spearman correlation of 0.96 was obtained (Fig. 4D).

The high spearman correlation demonstrated that within image sets, our label-free method correlates well, but the images have a very wide range of cell densities even with images that only have 10 cells per image. To get a better understanding how the analysis performed at different cell densities, we calculated the relative error for each image, as defined by the difference between the estimated number of cells a BF image and the number of nuclei on a logarithmic scale. For all four cell lines, the relative error was lower than 10 % for most images (Fig. 4E–I). However, at low cell density the relative error could rise to 50 % (Fig. 4F). This overestimation of cell number at low cell density, likely due to background or imaging artefacts being recognized by our algorithm as foreground pixels, demonstrates the limitations of the algorithm. Thus we show that our approach can be used on four morphologically distinct cell types, distinct instrumentation, and depending on the cell line over a wide range of cell densities, but typically above 250–500 cells per image.

### 4.5 Spatial characterization of label-free images

**Fig. 5:**
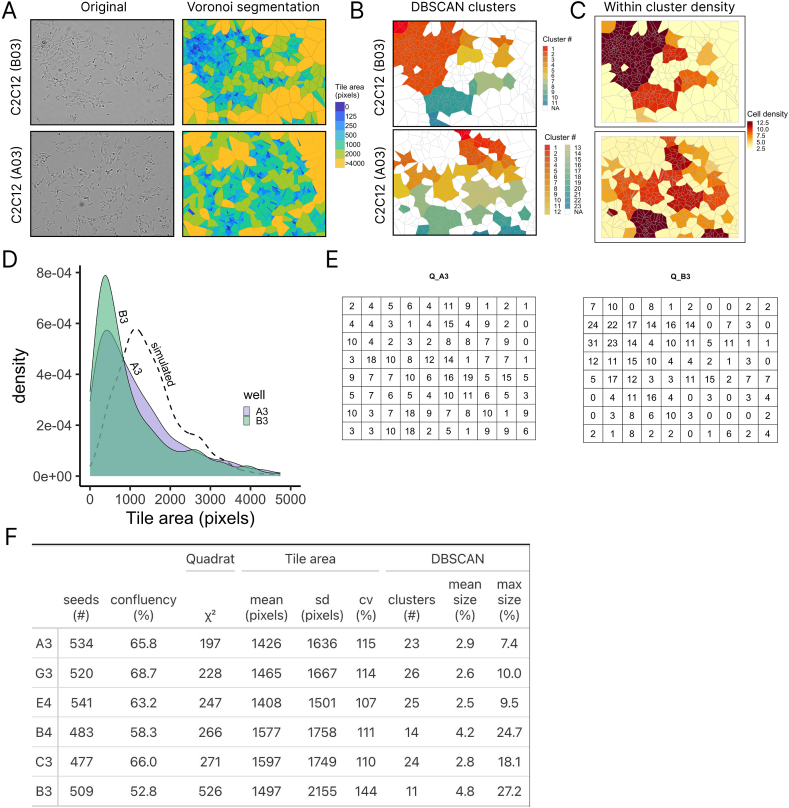
Spatial characterization of a set of C2C12 from multiple wells of a high-content imaging experiment. A) Representative images of a wells that were observed to either have cluster formation (well B03) or not (well A03). The Voronoi segmentation and tile coloring by tile size are also shown for each. B) Results of DBSCAN cluster analysis on the generated optimal seed points for each image. Tiles are colored by cluster number. Tiles that did not belong to a cluster according to DBSCAN or where not identifies as containing cells with the mixture modeling for not labeled. C) Coloring of identified clusters as in B by ‘within cluster density’ determined as number of tiles per cluster. D) Tile area density distribution of Voronoi segmented image of well B03 and A03. The dashed line represents a representative simulated seed point dataset. E) Quadrat counts of ‘number of seeds’ per area. Where the area is defined by a grid of 10 × 8 of well A03 (Q_A3) and well B03 (Q_B3). F) Summary table of spatial characteristics of six C2C12 images from the same plate with similar cell counts (500±50).

Apart from cell number quantitation, the xy positions of individual cells are approximated by our label-free image processing procedure, thus providing opportunities for spatial characterization of images. We compared six images from different wells of a C2C12 high-content imaging experiment in 96 well plates that were plated at similar density and had similar number of seeds (500±50). As an example, we first demonstrate the comparison between two wells of the C2C12 experiment. Transforming the original brightfield images of well B03 and A03 using Voronoi segmentation and coloring by tile size showed that well B03 had a relatively large cluster in the upper left corner of the image, whereas in well A03 the cells were more evenly distributed (Fig. 5A). We used the DBSCAN algorithm to identify how the seed points are clustered. Since DBSCAN does not require to specify the number of clusters on fore-hand, the number of clusters identified in an image can vary. For well B03, DBSCAN classified the seed points into 11 clusters (using eps = 40 and minPts = 5), whereas well A03 was classified into 23 clusters. When we combined the Voronoi segmentation polygons for each image with the cluster classification by coloring the tiles with cluster classification, one relatively large cluster was identified that overlapped with cells in the top left corner (Fig. 5B). Also neighbouring clusters were identified, that were distinct form the largest cluster in B03 (Fig. 5B). On the other hand, clusters in well A03 were smaller in size than in B03, also demonstrating a more even distribution. Furthermore, not only did well B03 have one particularly large cluster, also the relative within cluster cell density in that large cluster was high, indicating that the cells in well B03 where more concentrated into that particular cluster than in for example C03 (Fig. 5C).

Apart from the cluster analysis, we found that the distribution of all Voronoi derived tile sizes, could also provide spatial information about the cell spreading over the well. First, we simulated random uniform spatial point patterns, followed by performing the Voronoi segmentation on one xy coordinate set, and plotting the distribution (dashed line in Fig. 5D). A uniform distribution typically was less skewed to small tile areas, but also had asymetric distributions. Both the tile area distributions of well B03 and A03 peaked at a similar tile area, but the B03 distribution was more steep than the distribution of well A03 (Fig. 5D). Furthermore, we tested the seed points for Complete spatial randomness (CSR) using the quadrat. count function from the R spatstat package which counts the number of seeds within one quadrat of a grid (in our case a 10 × 8 grid) (Fig. 5E), followed by the quadrat. test function that assesses CSR using Chi-squared statistics. The sum of the Chi-squared per quadrat can give an indication on the magnitude of clustering of the seed points. By doing this for all six images in our dataset, we found that well A03 had the lowest Chi-squared (197), and well B03 had the highest Chi-squared (526) (Fig. 5F). Together with all other parameters from both the DBSCAN clustering analysis (number of clusters, mean cluster size and max cluster size), and the tile area distribution (mean, sd and cv of tile areas and confluency), we were able to demonstrate that using the xy coordinates from the label-free images we can generate a spatial parameter set that characterizes each image. We show that even though the number of cells in the image are nearly identical (500±50), the images are characterized by different spatial parameters.

## 5 Discussion

In this study, we describe a simple imaging processing pipeline for cell quantitation and spatial characterization of label-free images that are generated by high-content microscopy instruments. The tool is generalizable to multiple distinct cell types, and is implemented fully in R to provide the image processing and statistical computation in a single pipeline. Although deep neural network-based methods have been shown to segment cells, those methods require a large amount of training data sets with intensive manual annotation. In contrast, the method described here does not require training data and parameter optimization by users. By combining classical imaging processing procedures with Voronoi segmentation, Gaussian mixture modeling and automatic parameter optimization, our method can accurately estimate cell number (over 1000-fold magnitude) based on a single brightfield image. In addition to the cell number quantification, our tool can also be used to determine the spatial distribution of cells in the 2D plane. This feature is useful for quality control in cellular experiments and could be used to correlate cellular physiologic parameters with spatial distribution of cell populations.

Performing cell number quantification and spatial distribution assessment are routine tasks for any cellular experiment. In many labs, these daily activities rely on either manual assessment, commercial software packages, or imageJ and CellProfiler analysis. In multiple analysis pipelines, user input is needed, for example, for setting the threshold to distinguish background from foreground signals. This is also the case for other open source software packages, like Cellpose [22] and Ilastik [26]. Both tools require a human-in-the-loop workflow where the user needs to choose the best segmentation settings manually. In case of point-and-click software, this can limit the transparency and reproducibility of image analysis. Indeed, efforts have been made to standardize reporting of image analysis pipelines to improve reproducibility [5,27,28]. Our tool relies only on a single image and parameters are automatically optimized without user configuration. Combined with the open-source pipeline, this can ease the day-to-day image analysis workflow and improve the reproducibility of the image analysis process. In the past, such unsupervised workflows have been implemented for analyzing super-resolution microscopy images [[29], [30], [31]] and more recently, for calibration and segmentation of spatial transcriptomics data [32,33].

In this study, we analyzed brightfield images that were generated by automatic routine image capturing platforms, we did not test other label-free images generated by other image modalities, like DIC, QPI, raman microscopy, or electron microscopy. These imaging modalities generate images that have been shown to be able to be quantified for signals on the cellular level, as well as on the intracellular level [[1], [2], [3],^34^]. For example, using spinning-disk microscopy imaging of cells in culture combined with deep learning algorithms, Sun et al. [16] was able to predict intracellular structures based solely on brightfield images. Also, fibrin polymerization in fibrin clots was quantified using spatial light interference microscopy combined with mathematical modeling [35]. In addition, quantitative volumetric Raman imaging (qVRI) provides images of cellular specimen in 3D using raman scattering can provide quantitative information on lipid composition on the single-cell level, from label-free images [34]. These techniques hold promise for the future and will benefit from higher throughput and becoming more available for routine cell biology labs and facilities. Furthermore, the analysis of the images that are generated by these technologies could benefit from the automated iterative analysis that we used in our pipeline to be able to perform image segmentation with minimal user intervention.

One of the limitations of our tool is that it can only handle 2D images of cells in a plane and not 3D cell systems. Future studies could explore the possibility of Voronoi segmentation approaches in 3D applications of cultured cells. Another limitation of our tool is that accuracy of cell quantitation can be lower than when using other imaging segmentation approaches that rely on training datasets or more user interaction. Especially at low cell density (<250–500 cells per image) the tool overestimated the number of cells. This limit can be accounted for by using background images that do not contain cells. Since low cell density images are more likely to have background artefacts, speckles or imaging artefacts, the cell count can more easily be overestimated. Lab automation tools, like the one described in this study, often have some trade-offs between accuracy on the one side and practicality and time/resource investment on the order side. Our tool offers a good-enough estimate of cell number to be used in high-content imaging and automation. To improve the cell number estimate, multiple replicates of images can be analyzed to get a more accurate mean cell number estimate.

## 6 Conclusion

We generated an automatic image processing pipeline in R for cell number quantification and spatial characterization of label-free images. It requires less user intervention than current existing segmentation tools and does not require training data sets. The label-free image cell counts matched the ground truth nuclei counts for four different and distinct cell types. Combined, the open-source tool offers fast and reliable analysis of label-free images for routine use with high-content microscopic image datasets.

## CRediT authorship contribution statement

**Vincent C.J. de Boer:** Writing – review & editing, Writing – original draft, Visualization, Validation, Software, Resources, Project administration, Methodology, Investigation, Funding acquisition, Formal analysis, Data curation, Conceptualization. **Xiang Zhang:** Writing – review & editing, Writing – original draft, Validation, Resources, Methodology, Investigation, Formal analysis, Data curation, Conceptualization.

## Ethical statement

This study did not engage in human or animal testing.

## Data and code availability statement

All code and data are openly available with a CC BY 4.0 international license. R code and functions are deposited at https://github.com/vcjdeboer/segment_me_label_me, and data sets are available from Zenodo (https://zenodo.org/doi/10.5281/zenodo.11191022).

## Declaration of competing interest

The authors declare the following financial interests/personal relationships which may be considered as potential competing interests: Vincent de Boer reports financial support was provided by 10.13039/501100004890Wageningen University & Research. Xiang Zhang reports a relationship with AstraZeneca that includes: employment. If there are other authors, they declare that they have no known competing financial interests or personal relationships that could have appeared to influence the work reported in this paper.
